# New generation oral anticoagulant apixaban enhances embryo implantation by increasing integrin β3 expression in rats: A pilot study

**DOI:** 10.5935/1518-0557.20210122

**Published:** 2022

**Authors:** Sule Yildirim Kopuk, Nida Ozer, Yasemin Cekmez, Aslı Cakir, Kiran Gurkan

**Affiliations:** 1 Acibadem Maslak Hospital Assisted Reproductive Technologies Unit, Istanbul, Turkey; 2 Department of Obstetrics and Gynecology, Health Sciences University Umraniye Medical and Research Hospital, İstanbul, Turkey; 3 School of Medicine, Department of Pathology, Istanbul Medipol University, Istanbul, Turkey; 4 Department of Obstetrics and Gynecology, Bezmialem Vakif University, Istanbul, Turkey

**Keywords:** apixaban, anticoagulant, endometrial receptivity, integrin β3, implantation

## Abstract

**Objective:**

The first aim of this study was to investigate the effect of apixaban on endometrial receptivity via immunohistochemical investigation of integrin β3 expression in pregnant rats. The second aim was to compare the endometrial effects of both subcutaneous and oral anticoagulant drugs in terms of integrin β3 expressions.

**Methods:**

A total of 24 rats were selected for this study and divided into three equal groups as control, enoxaparin and apixaban groups. Subcutaneous enoxaparin and oral apixaban were applied for 15 days starting on the first day of pregnancy. On the 15th day of pregnancy, all rats were killed by cervical dislocation, and uterine horns, including pregnancy materials, were investigated for pregnancy success and endometrial receptivity by using immunohistochemical integrin β3 staining.

**Results:**

Living, viable fetuses were higher in the apixaban group compared to the control group (p=0.037). Intensity and universality of immunohistochemical staining of integrin β3 for endometrial stroma were detected statistically higher in the apixaban group than the other groups. (*p*=0.009 for intensity, *p*=0.014 for universality). Endometrial epithelial and myometrial integrin β3 expression were detected to be identical between the groups (*p*=0.3).

**Conclusions:**

Apixaban enhances endometrial receptivity via increasing integrin β3 expression in rats. This result can lead to further studies to be done in the future.

## INTRODUCTION

Embryo implantation is the most crucial step in the reproductive process to establish a successful pregnancy, and it requires a synchronized interaction between a receptive endometrium and healthy embryonic tissues (de Mouzon *et al*., 2010). Various markers have been described in the literature to better understand the mechanisms regulating embryo implantation in order to improve the ability of clinicians to treat infertility and prevent early pregnancy loss (Çekmez et al., 2016). Integrin β3 is a molecular marker of uterine receptivity in both humans and mice. Disruption of their expression have adverse effects on uterine receptivity and fertility (Dorostghoal *et al*., 2017). Its maximal expression happens on the surface of endometrial epithelium, coinciding with the time of implantation (Zhao *et al*., 2010).

Apixaban, a new generation oral anticoagulant, is a direct factor Xa inhibitor, which can be an alternative for low molecular weight heparin (LMWH) in case of their side effects, such as Heparin-Induced Thrombocytopenia (Chan *et al*., 2018; Khalid & Daw, 2017). Fixed drug dose without monitoring, fewer drug interactions, and a wide therapeutic window are advantages of the new generation oral anticoagulants (Mookadam *et al*., 2015). Although apixaban is widely used in cardiovascular system diseases, due to the increased risk of deep vein thrombosis in adults, its use in pregnant women is not as common as LMWH (Janczak *et al*., 2018; Robertson *et al*., 2015).

There are five pregnancy categories (A, B, C, D, X), defined by the degree to which available clinical and preclinical data rule out a risk for the fetus, according to the US Food and Drug Administration (FDA). The pregnancy category of apixaban has been reported as B, which means no evidence of risk to humans. Both animal studies show risk, but human findings do not; or, if no adequate human studies have been performed, animal findings are negative for risk (Boothby & Doering, 2001; Cada *et al*., 2013).

Although apixaban seems to be superior to LMWH in terms of an administration route and lesser side effects, there are currently no studies in the literature about the impact of the drug on embryo implantation. In this study, we aimed to investigate the effects of apixaban usage on endometrial receptivity in rats.

## MATERIALS AND METHODS

### Experimental animals

A total of 27 healthy rats with a body weight of 200 to 250g were taken from the Acıbadem University, Veterinary School - Animal Laboratory - after obtaining approval from the ethics committee of the same University (ACU-HADYEK 2017/20). The rats were fed routinely for one week before the experiment and were housed in a cage under standard laboratory conditions (22±2°C room temperature; 12-hour light/dark cycle and relative humidity of 55-50%). Tap water and food pellets were provided ad libitum throughout the experiment. Estrous female rats selected via the vaginal smear method were caged with male rats at a ratio of 1:1 overnight. The next morning, the female rats were individually assessed, and the day of detection of the vaginal plug or sperm-positive smear was designated as the first day of pregnancy. Three non-pregnant rats were excluded. We had 24 pregnant rats selected for the study, and they were divided into three equal groups: the LMWH group, the apixaban group, and the control group. We began to administer the drugs of each study group after the first vaginal plug detection. The drugs were applied as follows:

Control: No medication (Control)

LMWH group: enoxaparin 0.3mg/0.30 ml s.c. daily

Apixaban group: apixaban 0.25mg P.O. daily.

Acute toxicity studies were not conducted, because these doses were determined based on previous studies investigating fetal effects of these drugs in pregnancy; and the appropriate doses were determined in line with the findings from those studies (Wang *et al*., 2011; Figueiró-Filho *et al*., 2014).

The rats were slaughtered on gestational day 15, and then median laparotomy was performed. The uterine horns, including pregnancy material, were excised and stored in 10% formaldehyde. All the 1.5 mm and 3 mm cores of tissue array specimens were embedded in paraffin slices on coated slides, using the Immunohistochemical technique. They were washed in xylene to remove the paraffin, rehydrated through serial dilutions of alcohol, followed by washings with a solution of PBS (pH 7.2). All subsequent washes were buffered via the same protocol. The treated sections were then placed in a citrate buffer (pH 6.0) and heated in a microwave for three 5-minute sessions. The samples were then incubated with a monoclonal rat anti-Integrin beta-3 antibody (EPR2417Y, ab75872, Abcam, 1:150 dilution) for 60 minutes at 25°C. The conventional biotin-streptavidin method (Thermo, Ultravision anti-Polyvalent HRP/DAB Kit TP-015- HD, United States) was performed for signal development, and the cells were counter-stained with hematoxylin.

### Statistical Analysis

The data were analyzed using the SPSS software version 20.0 (SPSS Inc., Chicago, IL). Shapiro-Wilk test showed the data were not normally distributed; hence a nonparametric test, namely the Kruskal-Wallis test, was applied for further data analysis. For pairwise comparisons we ran the Dunn's post hoc test. The results were expressed as median, minimum and maximum. The categorical data were assessed with χ2 and Fisher Exact tests, as appropriate, and the values were expressed as numbers and percentages. *p*<0.05 was considered statistically significant.

## RESULTS

The mean numbers of total and living fetuses are listed in Table 1. Living fetuses were higher in the apixaban group than both the control and enoxaparin groups (*p*=0.037). The subgroup analysis performed showed a similar number of living fetuses in the apixaban and enoxaparin groups.

**Table 1.** Comparison of fetuses and living fetuses between the groups.

	Control (n=8)	Enoxaparin (n=8)	Apixaban (n=8)	*p* value
Number of fetuses Median (min-max)	7.5 (5-9)	8.5 (7-13)	9.5 (7-12)	0.071
Number of living fetuses Median (min-max)	6.5 (5-8)^a^	8 (6-12)^b^	8.5 (6-12)^b^	0.037

a,b b is statistically different from a.

**Figure 1:**
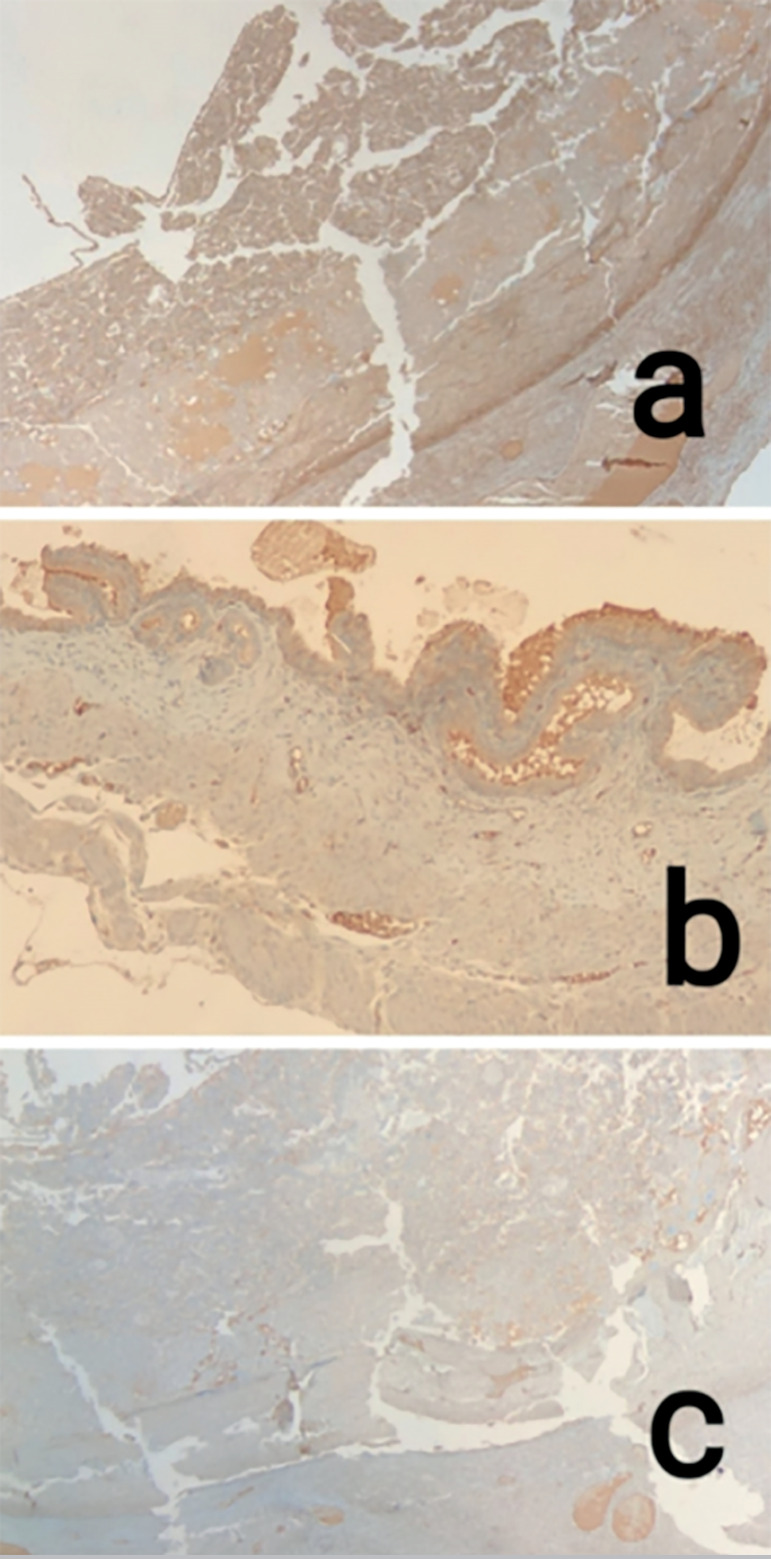
The immunohistochemical staining pictures of integrin β3 from the study groups. Intensity and universality of integrin β3 seems to be more in the apixaban group than others. a: apixaban group; b: enoxaparin group; c: control group.

Intensity and universality of immunohistochemical staining of integrin β3 for endometrial stroma were detected, and were statistically higher in the apixaban group, when compared to the other groups (*p*=0.009 for intensity, *p*=0.014 for universality) (Figure 1). According to the subgroup analysis, the intensity and universality of immunohistochemical staining of integrin β3 for endometrial stroma was significantly higher in the apixaban group when compared to the enoxaparin group, respectively (*p*=0.01 *vs*. *p*=0.01).

There was no statistically significant difference detected in the intensity and universality of immunohistochemical staining in the endometrial epithelium (*p*=0.642 for intensity, *p*=0.51 for universality) and myometrium (*p*=0.082 for intensity, *p*=0.131 for universality) among the groups (Table 2).

**Table 2.** Loboob Ingredients and their families, local name, used parts, percentages, main constituents and voucher numbers. Proportion of each ingredient showed in percentage.

	Control (n=8) (%)	Enoxaparin (n=8) (%)	Apixaban (n=8) (%)	*p* value
**Endometrial epithelium**				
- *Staining intensity*				0.642*
Absent	0 (0.0%)	0 (0.0%)	0 (0.0%)	
Light	6 (75%)	6 (75%)	4 (50%)	
Dark	2 (25%)	2 (25%)	4 (50%)	
- *Staining universality*				0.51
Absent	0 (0.0%)	0 (0.0%)	0 (0.0%)	
≤50%	2 (25%)	3 (37.5%)	1 (12.5%)	
>50%	6 (75%)	5 (62.5%)	7 (87.5%)	
**Endometrial stroma**				0.009*
-*Staining intensity*				
Absent	5 (62.50%)	7 (87.5%)	1 (12.5%)	
Light	2 (25%)	1 (12.5%)	7 (87.5%)	
Dark	1 (12.5%)	0 (0.0%)	0 (0.0%)	
- *Staining universality*				0.014*
Absent	5 (62.5%)	7 (87.5%)	1 (12.5%)	
≤50%	3 (37.5%)	1 (12.5%)	7 (87.5%)	
>50%	0 (0.0%)	0 (0.0%)	0 (0.0%)	
**Myometrial**				0.082*
-*Staining intensity*				
Absent	7 (87.5%)	4 (50%)	2 (25%)	
Light	1 (12.5%)	4 (50%)	5 (62.50%)	
Dark	0 (0.0%)	0 (0.0%)	1 (12.5%)	0.131*
- *Staining universality*				
Absent	7 (87.5%)	4 (50%)	2 (25%)	
≤50%	1 (12.5%)	3 (37.5%)	4 (50%)	
>50%	0 (0.0%)	1 (12.5%)	2 (25%)	

* Fisher’s exact *p *value.

## DISCUSSION

Integrins are adhesion molecules present on the endometrium, decidua, and extravillous cytotrophoblasts. They are expressed and play a crucial role during the window of implantation, and they also reflect endometrial receptivity. The reason for selecting integrin β3 as an endometrial receptivity marker by the authors is based on the current data in the literature, which confirm the importance of integrins in implantation. A number of studies demonstrated attenuated expression of integrin avβ3, and integrin β3 or a1 subunits in infertile women, and in women with endometriosis during the mid-secretory phase (Dorostghoal *et al*., 2017; Ivanov *et al*., 2010; Lessey, 2002). Animal studies also indicated a reduction in implantation following a functional blockade of integrin avβ3 (Liu *et al*., 2013; Illera *et al*., 2000).

A previous study reported that at the beginning of pregnancy, the change in integrin expression is synchronized with the trophoblast attachment; and αvβ3 integrin is expressed in the glandular epithelium during the window of implantation; and it translocates into endometrial stroma if pregnancy occurs (Lessey *et al*., 1994). According to the results of our study, intensity and universality of immunohistochemical staining of integrin β3 for endometrial stroma were considered statistically higher in the apixaban group when compared to the other groups (*p*=0.009 for intensity, *p*=0.014 for universality), similar to the data of related studies. The number of living fetuses were also higher in the apixaban group, compared to the control group, further supporting these results. There was no significant difference between the apixaban and enoxaparin groups, regarding the number of total and living fetuses.

The new generation oral anticoagulants provide direct inhibition of either thrombin (factor IIa; FIIa) or activated factor X (FXa). Their use is progressively rising around the world, as these new agents replace the historical anticoagulants, such as heparin and vitamin K antagonists, including warfarin, for various clinical conditions in medical practice (Krumme *et al*., 2018). Apixaban is one of the currently available new generation oral FXa inhibitor with Pregnancy Category B (Cada *et al*., 2013). The greatest advantage of apixaban is convenience, since there is no need for routine laboratory monitoring and frequent dose adjustments, as well as the reduced risk of intracranial hemorrhages (Schulman, 2014).

A subset of pregnant patients requires anticoagulation before and/or during pregnancy, including women at high risk of deep vein thrombosis, women with prosthetic heart valves, atrial fibrillation, cerebral venous sinus thrombosis, left ventricular dysfunction, and some women with fetal loss (Mardy *et al*., 2017; Fogerty, 2017; Cousin *et al*., 2018; James, 2018). Apixaban may be the first option that comes to mind in the presence of medical conditions that require the use of anticoagulants for pregnant patients or for patients planning pregnancy due to its fixed dose without the need for monitoring, few drug interactions and wide therapeutic window. According to the results of the present study confirming increased pregnancy rates and the number of live fetuses by using apixaban, the authors suggest using the drug in clinical situations, where anticoagulation is necessary.

According to our literature search of MEDLINE for articles in the English language with the terms 'endometrial receptivity', 'new generation oral anticoagulant' and 'integrin β3 expression' revealed no entries. As such, this study is perhaps the first in the literature to evaluate the effects of apixaban use on endometrial receptivity in pregnant rats. Subject number may be seen as a limitation of the present study, but power analyses revealed that to achieve 80% statistical power in the current study with an alpha level of 0.05, a minimum of 8 subjects were needed.

In conclusion, this study reports the increase of pregnancy rates by enhancing integrin expression in relation to apixaban usage. We hope that further investigations in this this will ensue.
